# A Predictive Model of Cardiovascular Aging by Clinical and Immunological Markers Using Machine Learning

**DOI:** 10.3390/diagnostics15070850

**Published:** 2025-03-27

**Authors:** Madina Suleimenova, Kuat Abzaliyev, Madina Mansurova, Symbat Abzaliyeva, Almagul Kurmanova, Guzel Tokhtakulinova, Akbota Bugibayeva, Diana Sundetova, Merei Abdykassymova, Ulzhas Sagalbayeva, Raushan Bitemirova, Zhadyra Yerkin

**Affiliations:** 1 Department of Big Data and Artificial Intelligence, Faculty of Information Technology, Al-Farabi Kazakh National University, Almaty 050040, Kazakhstan; mansurova.madina@gmail.com (M.M.); abzaliyeva.symbat@gmail.com (S.A.); erkinjadyra02@gmail.com (Z.Y.); 2 Department of Internal Medicine, Faculty of Medicine and Healthcare, Al-Farabi Kazakh National University, Almaty 050040, Kazakhstan; abzaliev_kuat@mail.ru (K.A.); almagul.kurmanova@kaznu.edu.kz (A.K.); bota_88.20@mail.ru (A.B.); sdiana92@mail.ru (D.S.); merei1808@mail.ru (M.A.); ulya_sagalbayeva@mail.ru (U.S.); r.bitemir@gmail.com (R.B.); 3 Department of Strategic Research, Science Center of Obstetrics, Gynecology and Perinatology, Almaty 050020, Kazakhstan; guzel-733@mail.ru

**Keywords:** artificial intelligence, machine learning, cardiovascular aging, predictive modeling, immunological markers

## Abstract

**Background/Objectives:** Aging and immune mechanisms play a key role in the development of cardiovascular disease (CVD), especially in the context of chronic inflammation. Therefore, in order to detect early aging in the elderly, we have developed a prognostic model based on clinical and immunological markers using machine learning. **Methods:** This paper analyzes the relationships between immunological markers, clinical parameters, and lifestyle factors in individuals over 60 years of age. A machine learning (ML) model including random forest, logistic regression, k-nearest neighbors, and XGBoost was developed to predict the aging rate and risk of CVD. Correlation anal is revealed significant associations between immune markers (CD14+, HLA-DR, IL-10, CD8+), clinical parameters (BMI, coronary heart disease, hypertension, diabetes), and behavioral factors (physical activity, smoking, alcohol). **Results:** The results of the study confirm that systemic inflammation, as reflected by markers such as CD14+, HLA-DR, and IL-10, plays a central role in the pathogenesis of aging and related diseases. CD14+ shows a moderate positive correlation with post-infarction cardiosclerosis, accounting for 37%. HLA-DR correlates with body mass index at 39%. A negative association between IL-10 level and BMI was also found, where the correlation reaches 52% (r = −0.52). The level of CD8+ cells shows a negative correlation with smoking and their number, being 40%. Training was performed on clinical and immunological data and models were evaluated using accuracy, ROC-AUC, and F1-score metrics. Among all the trained models, the XGBoost model performed best, achieving an accuracy of 91% and an area under the ROC curve (AUC) of 0.8333. **Conclusions:** The study reveals significant correlations between immunological markers and clinical parameters, which allows the assessment of individual risks of premature cardiovascular aging. R (version 4.3.0) and specialized libraries for correlation matrix construction and visualization were used for data analysis, and Python (version 3.11.11) was used for model development and training.

## 1. Introduction

Population aging and increasing life expectancy led to a significant increase in age-related diseases, especially cardiovascular disease (CVD), which remains the leading cause of death worldwide. In recent years, the role of the immune system in these processes has attracted the attention of researchers, with immunosenescence and inflammatory senescence (inflammation) being the main mechanisms. Immunosenescence is associated with age-related changes in the composition and function of immune cells such as T- and B-lymphocytes, resulting in a weakened immune response, increased susceptibility to infections, and an increased risk of chronic inflammatory diseases.

Studies [1] show that with age, there is a steady increase in pro-inflammatory cytokines such as interleukin-6 (IL-6) and tumor necrosis factor alpha (TNF-α), which stimulate systemic inflammation leading to rapid cardiovascular aging. These processes play a crucial role in the pathogenesis of diseases such as atherosclerosis and coronary heart disease (CHD), exacerbating vascular damage and preventing normal tissue repair.

The aim of this work is to develop a predictive model based on clinical and immunological markers using machine learning to detect early cardiovascular aging. Correlation matrices revealed key relationships between immune activity, lifestyle, and clinical parameters. Current studies demonstrate a significant correlation between immune-inflammatory processes and age-associated diseases, but the mechanism of the relationship requires further investigation [2,3].

Scientific advances in recent years, in particular the development of artificial intelligence (AI) techniques, are opening new opportunities for analyzing and interpreting biomedical data. Artificial intelligence techniques, including machine learning and big data analytics, can identify complex relationships between biomarkers, clinical indicators, and environmental factors that provide a personalized approach to diagnosis and prognosis [4].

## 2. Literature Review

Personalized prediction of cardiovascular disease using multi-omics technologies and machine learning techniques was conducted in a study [5] that combines RNA sequencing data, single nucleotide polymorphisms (SNPs), and clinical information to create personalized risk profiles. Using trait selection methods, researchers identified 27 key transcriptomic markers and SNPs that distinguish patients with cardiovascular disease. An optimized XGBoost model tuned with Bayesian hyperparameters demonstrated high accuracy. Risk assessment using Shepley’s additive mixture helped to explain the importance of biomarkers (RPL36AP37 and HBA1).

Dipa, R., Sadu et al. applied machine learning to predict CVD based on electronic health records including biomarkers such as cholesterol levels, blood pressure, body mass index, glucose levels, and family history [6]. Using logistic regression, decision tree, SVM, and neural network methods, the authors optimized data processing by normalizing and removing outliers, which improved the accuracy of the models. The results confirm the effectiveness of the algorithms in predicting CVD. Further research aims to improve personalized prevention approaches.

Another important contribution was made by Liu, Z. S. et al. [7], who investigated NHS incidence among people with HIV (PLHIV) using South Carolina electronic health records data. The study included 9082 patients diagnosed with HIV between 2006 and 2019. Multivariate logistic regression models were used to analyze the association between traditional risk factors and cardiovascular disease. Forest plots were used to visualize adjusted odds ratios (aOR) and confidence intervals. The results showed that smoking, hypertension, obesity, and chronic kidney disease were consistently associated with cardiovascular disease, while the effects of viral suppression decreased with increasing follow-up time, and the results emphasize the importance of optimal viral suppression in the short term and restoration of immune function to reduce cardiovascular disease risk in the long term.

A large-scale study [8] used data from 10,432 individuals aged 40 to 69 years to compare 17 measures of adiposity (ROC) to predict arterial hypertension and dyslipidemia, which are major predictors of cardiovascular disease, type 2 diabetes (T2DM), and multimorbidity. ROC and logistic regression curves showed that the Chinese visceral adiposity index (CVAI) and triglyceride-glucose index (TYG) better predicted arterial hypertension and multimorbidity, and body mass index (BMI) better predicted dyslipidemia.

An assessment of 10-year cardiovascular disease (CVD) risk using the WHO scale is presented in a study [9] using data from 5503 adults in Malaysia. It was found that 4.9% of participants had high risk (≥20%), more common in males (7.3%) compared to females (2.5%). Major risk factors included low education level, unemployment, and obesity combined with physical inactivity (aOR = 2.19). Recommended interventions to reduce risk were health promotion, screenings, and information campaigns. These results highlight the importance of targeted prevention programs for high-risk groups.

Currently, artificial intelligence (AI) is being actively introduced in cardiology, offering data analysis techniques for the diagnosis and treatment of cardiovascular disease (CVD) [10]. Deep learning models, including convolutional neural networks (CNNs) and recurrent neural networks (RNNs), have demonstrated high accuracy in ECG processing and disease prediction. For example, AI-ECG can effectively detect asymptomatic valve defects and atrial fibrillation, reducing the risks of underdiagnosis. The work highlights the potential of AI to improve diagnostic accuracy and develop affordable screening tools, especially in resource-limited settings. For example, a study by Liu, J. et al. examined the association between serum neurofilament light chain (sNFL) levels and cardiovascular disease (CVD) and assessed the prognostic value of sNFL. Using data from 2035 NHANES 2013–2014 participants, high sNFL levels were found to be associated with increased odds of CVD (OR = 1.41), especially in individuals without hypertension [11]. Adding sNFL to risk models improved predictive accuracy (AUC 0.845), especially in participants without hypertension. However, in individuals with hypertension, sNFL did not improve prognosis. The study highlights the potential of sNFL as a biomarker for the diagnosis and prognosis of CVD, especially in populations without hypertension, with the need for further longitudinal studies.

The study investigates cardiovascular disease (CVD), a leading cause of death worldwide and increasingly prevalent in low- and middle-income countries such as Bangladesh. Random forest was the best performing classifier, achieving 98.04% accuracy, 96.15% reliability, 100% completeness, and an AUC of 0.989. In comparison, logistic regression showed the lowest accuracy of 95.42%. The study highlights the random forest model as an effective tool for predicting CVD risk with potential implications for improving clinical decision making and improving patient prognosis [12].

An interesting study was conducted using a machine learning (ML) model developed to predict cardiovascular disease (CVD) in Korean patients with type 2 diabetes mellitus (T2DM). Using data from detection (*n* = 12,809) and validation (*n* = 2019) cohorts collected between 2008 and 2022, the study evaluated different ML models with hyperparameter adjustment. Random forest (RF) showed the best performance, achieving an AUROC of 0.830 (95% CI: 0.818–0.842) in the detection dataset and 0.722 (95% CI: 0.660–0.783) in the validation dataset. Creatinine and glycated hemoglobin levels were identified as key predictors in the RF model. The results show that this ML-based model offers an excellent method for predicting CVD in patients with DM2, paving the way for early personalized preventive strategies [13].

An epidemiological study of cardiovascular disease (CVD) in Kashgar Prefecture, Xinjiang, northwest China was conducted to identify key risk factors. Data from 1,887,710 adults (baseline 2017) from the Kashgar prospective cohort study were analyzed, including 16 potential factors—7 demographic, 4 lifestyle, and 5 clinical factors—collected through questionnaires and medical examinations. Logistic regression models showed that all factors were significantly associated with CVD (odds ratio 1.03 to 2.99, *p* < 0.05). Machine learning methods (random forest, random ferns, extreme gradient boosting) ranked age, occupation, hypertension, exercise frequency, and dietary patterns as the five major risk factors for CVD. This study considers cardiovascular disease as a major problem in Kashgar and these five factors are crucial for future preventive measures [14].

The aim of a study conducted in East China was to develop a cardiovascular disease (CVD) prediction model to estimate the 3-year risk of CVD among high-risk individuals. Logistic regression identified about 30 factors associated with CVD, such as male gender, older age, family income, smoking, alcohol consumption, obesity, abnormal cholesterol, and blood glucose levels. Several machine learning methods were tested including multivariate regression, CART, naive Bayes, bagged trees, AdaBoost, and random forest. The random forest model outperformed the others, achieving an AUC of 0.787, which significantly improved the multivariate regression benchmark (AUC = 0.7143). The study provides a robust prediction model for CVD using the random forest algorithm, offering valuable information for CVD prediction and treatment in China [15].

The importance of model accuracy in predicting cardiovascular disease risk was emphasized in a study that aimed to improve cardiovascular disease (CVD) prediction and diagnosis using data mining and machine learning (ML) techniques [16]. The study applied both supervised and unsupervised MO techniques including k-NN, SVM, random forest, artificial neural network (ANN), naive Bayesian algorithm, logistic regression, stochastic gradient descent (SGD), and AdaBoost. Unsupervised clustering methods such as k-means, hierarchical, and density-based spatial clustering were used to identify clusters in the data. The results showed that SGD and ANN models provided the highest classification accuracy with a score of 0.900.

A machine learning-based approach to diagnose cardiovascular disease (CVD) using the ‘Cardiovascular Disease Dataset’ from Kaggle, was used by Mridha K. Six machine learning algorithms were tested, with logistic regression achieving the highest accuracy of 74%. Cross-validation was applied to further improve the accuracy, resulting in an increase in accuracy to 84.53%. The study also utilized SHAP and LIME interpretability methods to identify the most influential features contributing to the model predictions. This approach provides a promising solution for the diagnosis of SWD, combining high accuracy with interpretability, and allowing healthcare professionals to make informed decisions [17].

Interesting findings have been obtained using the modified artificial bee colony (M-ABC) algorithm combined with k-nearest neighbors (k-NN) for optimal feature selection. Using the UCI Cleveland heart disease dataset, the authors used preprocessing techniques such as direct coding and Z-score normalization along with the SMOTE technique to eliminate class imbalance. The M-ABC algorithm, enhanced with the Firefly algorithm, optimizes feature selection, while the k-NN classifier provides accurate predictions. The framework achieved excellent performance metrics including 89.7% accuracy, 85.6% sensitivity, and 94.8% specificity, outperforming traditional methods such as genetic algorithm with SVM/k-NN and M-ABC with SVM. Implemented in Python with 10-fold stratified cross-validation, the approach is computationally efficient and privacy-aware using IoMT-based systems. The study highlights its potential for future research on adapting this framework to other diseases using advanced machine learning techniques [18].

In conclusion, scientific advances in recent years, in particular the development of artificial intelligence (AI) techniques, have opened new opportunities for analyzing and interpreting biomedical data. Many studies are conducted using machine learning techniques to establish relationships between risk factors, model and interpret complex clinical data, and predict outcomes with high accuracy. Artificial intelligence techniques, including machine learning and big data analytics, can identify complex relationships between biomarkers, clinical indicators, and environmental factors, providing a personalized approach to diagnosis and prognosis.

## 3. Materials and Methods

The study was conducted within the framework of the scientific and technical project №AP19677754 ‘Development of markers and diagnostic algorithm for detection and prevention of early cardiovascular aging’, State Institution “Ministry of Science and Higher Education of the RK”.

The study included 52 people aged 60 years and older, observed in polyclinics and medical centers in Almaty. By sex, there were 18 (35%) men and 34 (65%) women. The average age of men was 82.9 ± 10.0 years and the average age of women was 81.5 ± 10.0 years. The individuals included in the study were divided into 2 groups according to the presence of cardiovascular diseases. Group 1 included 30 participants who had a history of cardiovascular disease. The mean age of the elderly was 69.2 ± 3.1 years, senile 83.1 ± 4.0 years, and long-lived 93.6 ± 2.7 years. Group 2 included 22 individuals who had no history of and at the time of inclusion in the study reported heart and vascular disease. The mean age of elderly persons was 69.3 ± 2.1 years, senile persons 83.1 ± 2.6 years, and long-lived 92.2 ± 2.6 years. All study participants received complete, reliable, and objective information about the study and voluntarily and independently agreed to participate in the study.

The blood samples were collected and transferred during 2 h to the Scientific Center of Obstetrics, Gynecology and Perinatology. The samples were stained with monoclonal antibodies (mAb) using reagents from Becton Dickinson (BD) against CD4+, CD8+, CD16+, CD56+, CD14+, CD19+, CD95+, and HLA-DR for staining and binding of surface receptors; 5 µL of mAb was added to a tube with 50 µL of the sample of peripheral blood, mixed on a vortex and incubated for 15 min at room temperature in the dark. After incubation, lysing by BD harm Lyse Lysing Buffer, the mixture was vortexed and incubated for 10 min at room temperature in the dark. The mixture was centrifuged for 5 min at 300 g/min, after which the supernatant was removed. Membrane permeabilization was performed with Cytofix/Cytoperm solution, followed by mAb against TNF-α, GM-CSF, VEGFR-2, IPGF, and Perforin+ for staining and binding intracellular receptors. Immune status was investigated on a BD FACS CALIBUR (USA) flow cytometer using the CELL Quest program.

Exclusion criteria: HIV infection, known tuberculosis, acute infectious diseases within 3 months prior to inclusion, mental illness that limited adequate co-operation, diagnosed allergic reaction of any type, and refusal to participate in the study.

The aim of the study was to evaluate the prognostic and diagnostic performance of immunological assays in older adults with cardiovascular disease.

This study used correlation analysis to examine the relationships between immunological markers, clinical parameters, and lifestyle factors in patients with and without cardiovascular disease (CVD). The following biomarkers were used:

- CD95+, CD59+, CD16+, CD14+, CD19+, CD8+, CD4+: Markers of the activity and abundance of different types of immune cells such as T-lymphocytes, B-lymphocytes, and natural killer cells.
- IL-10: A cytokine associated with the anti-inflammatory response.
- IPGF: An indicator associated with growth factors.
- TNF-α: Tumor necrosis factor, a marker of inflammation.
- VEGF-2: Vascular endothelial growth factor, associated with angiogenesis.
- Perforin+: A protein involved in the destruction of infected cells.
- GM-CSF: Granulocyte-macrophage colony-stimulating factor associated with immune function.
- HLA-DR: Protein involved in antigen presentation.
- CD56+: Marker of natural killer cell activity.
  Clinical Indications:
- Arterial hypertension (AH).
- Acute cerebral circulatory disorders/stroke (ACVD).
- Body mass index, a measure of the degree of obesity (BMI).
- Diabetes mellitus (DM).
- Atrial fibrillation (AF).
- Chronic heart failure (CHF/IHD).
- Cerebro-vascular disease (CVD).
- Atrioventricular block (AV blockade).
- Presence of a coronary bypass or stent.
- Post-infarction cardiosclerosis (PICS).
  Lifestyle:
- Smoking: Presence of an unhealthy habit.
- Alcohol: Alcohol consumption.
- Physical activity: Level of regular physical activity.

Methods were used to construct and analyze several correlation matrices that visualized relationships between variables including biomarkers, clinical factors and behavioral parameters. Artificial intelligence (AI) techniques such as machine learning was applied in the study. Three different methods were chosen to build classification models: random forest, logistic regression and k-nearest neighbors method. These methods were chosen to study their performance and differences in classification approaches. Random forest is an ensemble method based on the construction of multiple decision trees to handle complex dependencies in the data. Logistic regression is used as a basic linear model well suited for binary classification tasks. The k-nearest neighbors method, on the other hand, is based on distances between objects, making it sensitive to the structure of the data. XGBoost, on the other hand, is one of the most powerful gradient boosting methods, which allows efficient handling of non-linear dependencies and shows high classification accuracy rates. Correlation matrices were constructed to identify linear relationships between the variables under study. The values of correlation coefficients ranged from −1 (complete negative correlation) to 1 (complete positive correlation), where 0 indicated no relationship. Visualization was conducted using a color scale where red represented positive correlations, blue represented negative correlations, and white represented values close to zero. The matrix analyzed significant correlations such as the positive correlation between HLA-DR and body mass index (r = 0.5), reflecting activation of the immune system in overweight individuals, and the negative relationship between CD8+ and smoking (r = −0.43), indicating suppression of the adaptive immune response in smokers. The inclusion of variables such as physical activity level, education, and chronic inflammatory diseases allowed us to identify their influence on immunological parameters and clinical status of the participants. For data analysis, R (version 4.3.0) and specialized libraries were used to construct correlation matrices and visualize them. Missing data (<8%) were filled by random forest using the missForest package.

In the next step, each of the models was trained on the data trained above. For each model, separate experiments were conducted with visualization of key metrics such as ROC curve and error matrix to evaluate the classification quality and identify the strengths and weaknesses of the receiver operating characteristic curve (ROC) and area under curve (AUC) approaches. The XGBoost model performed best, achieving AUC = 0.8333, confirming its high performance in predicting cardiovascular disease.

## 4. Results

**Figure 1:**
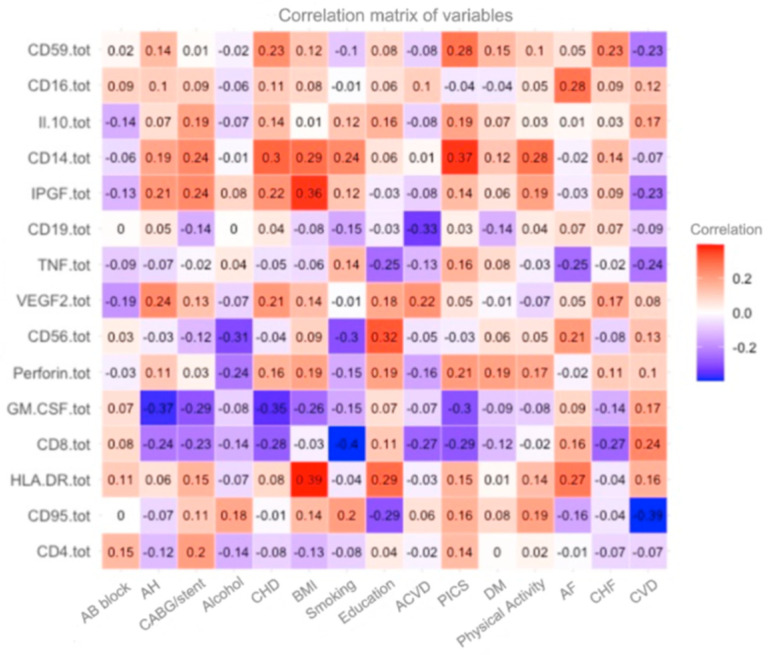
A general graph with all patients.

In this study, a correlation matrix (Figure 1) was constructed to reflect the relationships between immunological and clinical markers for analyzing risk factors associated with premature aging and related diseases. Based on the correlation matrix, where each cell represents the degree of correlation between two variables, significant patterns influencing biological aging and the development of chronic diseases can be identified.

The matrix includes the following variables:

- **Immunological markers**: CD59+, CD16+, CD14+, IL-10, IPGF, CD19+, TNF-α, VEGF-2, CD56+, Perforin+, GM-CSF, CD8+, HLA-DR, CD95+, and CD4+, which reflect the state of the immune system.
- **Clinical indicators**: Body mass index (BMI), the presence of cardiovascular diseases (e.g., CHD, hypertension, diabetes, etc.), as well as physical activity, smoking, and alcohol consumption.

The color scale of the matrix ranges from deep red (positive correlation) to blue (negative correlation), with white indicating neutral correlations close to zero. The numerical correlation values range from −1 to 1, where:

- **1** indicates a perfect positive correlation,
- **0** indicates no correlation,
**−1** indicates a perfect negative correlation.

The correlation matrix shown in the image visualizes the relationship between immunological parameters and various external factors or body conditions. This can be useful for understanding how different immune cells or inflammatory markers are related to clinical conditions, lifestyle, or physiological parameters [19].

The correlation between HLA-DR levels and BMI of 0.39 indicates a positive association between these measures. This may indicate that an increase in BMI is accompanied by an increase in HLA-DR expression, which in turn may reflect changes in immune activity in overweight individuals. This relationship suggests that obesity and associated metabolic disorders may contribute to the activation of the immune system, which is confirmed by the increased expression of HLA-DR as a marker of immune cell activation.

A positive correlation of 0.37 between CD14+ level and post-infarction cardiosclerosis (PIC) suggests an association between monocyte activity and the development of myocardial fibrotic changes after myocardial infarction. CD14+ is a marker of monocyte activation, which plays a key role in inflammatory processes and cardiac tissue remodeling. This correlation suggests that as CD14+ levels increase, the likelihood of more severe post-infarction cardiosclerosis increases. This may indicate that systemic inflammation, reflected by increased CD14+ levels, contributes to the progression of myocardial scarring and deterioration of myocardial function. Also, a study [20] determined the causal effects of HLA-DR-expressing CD14+ monocytes on SCD. This also proves that these features are biomarkers having influence in the immune system.

A good association (r = 0.3) was observed between IPGF (insulin-like growth factor) levels and coronary heart disease (CHD), indicating a possible link between this growth factor and the development of cardiovascular pathologies. IPGF plays an important role in the processes of cell proliferation, tissue regeneration, and angiogenesis, which may be related to vascular and myocardial remodeling in ischemic heart disease. This correlation may suggest that increased IPGF levels promote enhanced angiogenesis and vascular remodeling, which in the context of ischemic heart disease may reflect the body’s compensatory mechanisms for vascular injury.

An interesting association was observed between education and HLA-DR, suggesting a possible link between educational status and immune system activity reflected by HLA-DR expression. HLA-DR is a marker of activation of antigen-presenting cells, such as macrophages and dendritic cells, and plays a key role in the regulation of immune responses. This correlation may indicate that people with higher levels of education tend to have more active immune systems, which may be related to lifestyle, healthy habits, or access to health knowledge and services. In addition, education level is often associated with socioeconomic status, which can influence stress levels, nutrition, and physical activity, which in turn influence immune system health.

A negative correlation of −0.4 between CD8+ levels and smoking indicates an inverse relationship between CD8+ T-lymphocyte counts and smoking habit. CD8+ T-lymphocytes play a key role in the adaptive immune response, including killing virus-infected cells and controlling tumor cells. This negative correlation may indicate that smoking reduces the number or functionality of CD8+ T cells, which in turn may weaken the body’s immune response. Smoking is known for its negative effects on the immune system as it can suppress the activity of cells involved in protective immune responses and increase susceptibility to infections and inflammatory diseases. This result confirms that smoking has a negative impact on key components of immune defense, by reducing CD8+ T-lymphocyte levels, which may have long-term health consequences.

The negative correlation of −0.39 between CD95+ levels and chronic inflammatory diseases (CIDs) indicates an inverse relationship between CD95+ (also known as the Fas receptor) expression and the risk or severity of chronic inflammatory diseases. CD95+ plays a key role in the process of apoptosis by regulating programmed cell death. Increased expression of CD95+ may enhance cell apoptosis, which helps control excessive inflammation and limit tissue damage caused by chronic inflammatory processes. A negative correlation may indicate that patients with lower CD95+ levels have an increased risk or more severe course of chronic inflammatory diseases. This may be due to impaired apoptosis mechanisms that favor the accumulation of damaged or non-functional cells that support the inflammatory process.

On the contrary, increased CD95+ levels associated with more active apoptosis may contribute to a reduction in chronic inflammation, leading to better inflammatory control. Moreover, we see that CD95+ has no association with AV blockade, CD19+ with alcohol, and CD4+ with diabetes mellitus. The negative correlation may suggest that patients with lower CD95+ levels have an increased risk or more severe course of chronic inflammatory diseases. This may be due to impaired apoptosis mechanisms that favor the accumulation of damaged or non-functional cells that support the inflammatory process.

**Figure 2:**
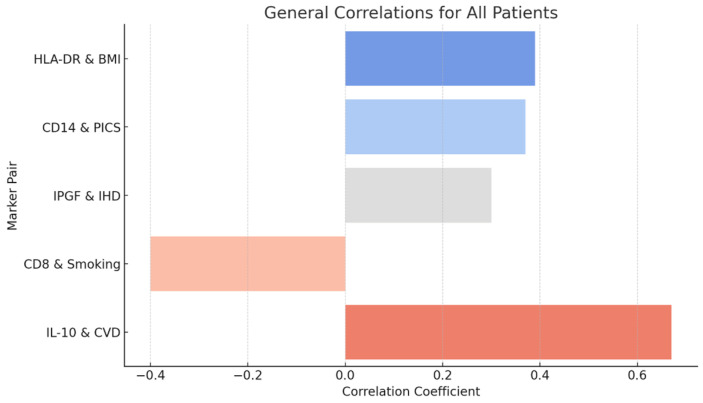
Correlations of immunological markers with clinical indicators in patients of all categories.

The graph shows (Figure 2) the key correlations between immunological and clinical markers in the general group of patients. These relationships can be interpreted in terms of pathophysiological processes associated with systemic inflammation, metabolic disorders, and risk factors for chronic diseases. These correlations confirm the importance of studying the interactions between immunological markers and clinical indicators for understanding the mechanisms of biological aging and the development of chronic diseases. Positive correlations may indicate pathophysiological changes associated with compensatory mechanisms or disease progression, whereas negative correlations demonstrate suppression of key immune functions under the influence of harmful factors.

**Figure 3:**
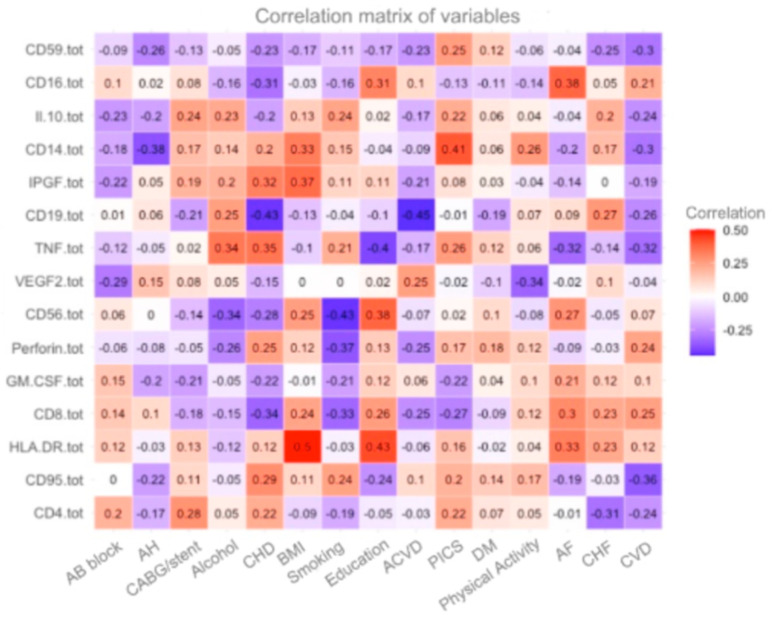
Patients with CVD.

Another correlation matrix is presented (Figure 3), visualizing the association between immunological parameters and clinical factors associated with cardiovascular disease (CVD). A positive relationship can be observed in HLA-DR and education (r = 0.43), as well as in the description above. The positive correlation indicates that people with higher education levels may have increased expression of HLA-DR, reflecting activation of antigen-presenting cells. This may be related to healthier lifestyles or greater health awareness among people with higher levels of education. Also, HLA-DR and BMI have a good correlation (r = 0.5). The positive correlation between CD14+ and PIC indicates that increased CD14+ levels are associated with increased risk or severity of post-infarction cardiosclerosis. This may indicate that systemic inflammation through monocyte activity (of which CD14+ is a marker) plays a role in the progression of cardiosclerosis.

The positive correlation of 0.38 between CD8+ T-lymphocyte levels and atrial fibrillation (AF) suggests that there is an association between cytotoxic T-cell activity and the presence or development of atrial fibrillation. CD8+ T lymphocytes play an important role in the immune response, including destruction of infected and abnormal cells, as well as participation in the regulation of inflammatory processes. This positive correlation may indicate that increased levels of CD8+ cells are associated with the development or progression of atrial fibrillation. Atrial fibrillation is often associated with inflammatory processes in the myocardium, and the immune system, particularly cytotoxic T-lymphocytes, may be involved in the maintenance of these processes. Thus, CD8+ cell activity may enhance or maintain inflammation in cardiac tissue, which may contribute to impaired electrical activity and the development of arrhythmias. This result emphasizes the importance of the immune system in the pathogenesis of atrial fibrillation and suggests that immunomodulation may be a potential avenue for the prevention and treatment of this condition.

The negative correlation of −0.43 between CD19+ cell levels and acute cerebral circulatory failure (ACF) indicates a significant inverse relationship between B-lymphocyte count and the risk or severity of ACF. CD19+ is an important marker of B-lymphocytes, which play a key role in the regulation of the adaptive immune response, especially in antibody production and immune memory. The negative correlation of −0.43 may mean that patients with reduced CD19+ B-lymphocyte levels have an increased risk of developing cancer or worsening after such events. This association may be because B-lymphocytes have an anti-inflammatory effect, helping to contain inflammation that can contribute to vascular damage and impaired cerebral blood flow. Decreased B-lymphocyte counts, as reflected by the correlation, may indicate an impaired immune response, which may contribute to vascular deterioration and increased risk of vascular incidents such as stroke. Thus, the negative correlation of −0.45 between CD19+ and ONMC emphasizes the possible protective role of B cells in the context of vascular disease.

Also, the negative correlation of −0.43 between CD19+ cell levels and coronary heart disease (CHD) may be related to the fact that B-lymphocytes have a modulating effect on inflammation, which plays a central role in the pathogenesis of atherosclerosis, the main mechanism of CHD development. A decrease in the number of B-lymphocytes can weaken the immune control of inflammatory processes, which can accelerate the development of atherosclerotic plaques and worsen the condition of blood vessels. This, in turn, increases the likelihood of developing coronary heart disease. Moreover, we can emphasize that in this figure we do not see an association between such biomarkers as CD56+ and AH, IPGF with CVD, and VEGF with BMI and smoking.

**Figure 4:**
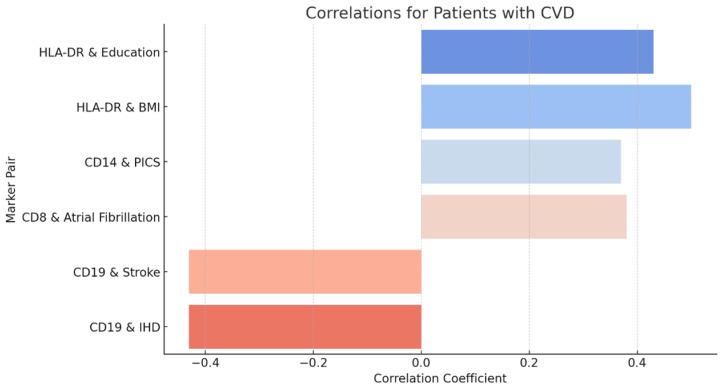
Correlations of immunological markers with clinical indicators in patients with CVD.

The graph (Figure 4) shows correlations between immunological and clinical parameters in patients with cardiovascular disease (CVD). The revealed correlations confirm the connection of activation of certain cell markers with the progression of metabolic, vascular and inflammatory disorders.

**Figure 5:**
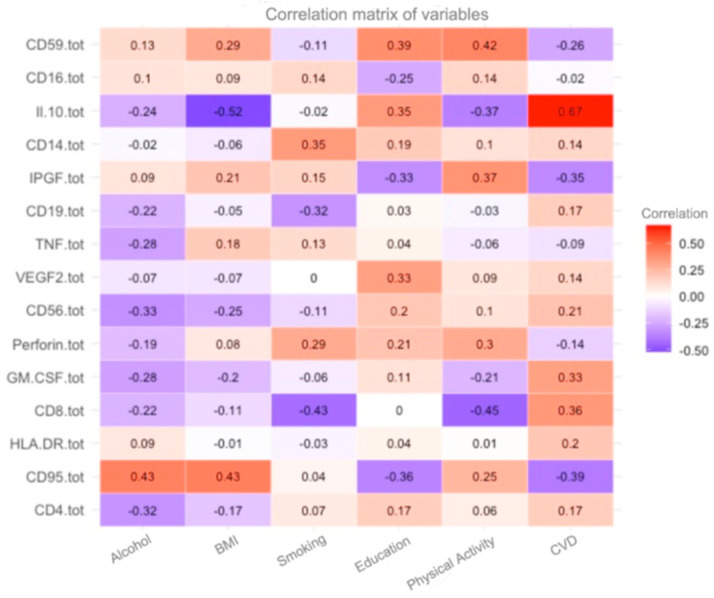
Patients without CVD.

And we also made a correlation matrix of patients without cardiovascular diseases (Figure 5):

A pronounced association is observed between IL-10 and CVD which is associated with more severe chronic inflammatory diseases. IL-10 is an important anti-inflammatory cytokine, and its increase may be the organism’s response to prolonged inflammation, which is characteristic of CHD (r = 0.67). We also observed an interesting positive correlation between CD95+ and alcohol and BMI, which is r = 0.43. This correlation may indicate that smoking and increased BMI are associated with increased expression of CD95+, a marker of apoptosis. This may indicate that apoptosis processes are enhanced in smokers and overweight people, which may be the result of chronic inflammation or cellular stress induced by these factors. Moreover, some indicators do not correlate with each other. For example, VEGF-2 with smoking and CD8+ with education. The low correlation value (close to 0) between these indicators indicates that there is no significant linear relationship between them. This means that changes in one indicator are not related to changes in the other.

However, according to the correlation matrix above, we see inverse relationships such as IL-10 and BMI (r = −0.52). This negative correlation may indicate that as BMI increases, the level of anti-inflammatory cytokine IL-10 decreases. This may indicate that anti-inflammatory mechanisms are suppressed in people with higher BMI, which may contribute to the increased systemic inflammation characteristic of obesity and associated metabolic disorders. CD8+ also had a negative association with physical activity and smoking (r = 0.45). This means that as physical activity increases and smoking decreases, CD8+ cell levels decrease. This relationship may indicate that smoking negatively affects the number or functionality of CD8+ cells, weakening the adaptive immune response. In contrast, physical activity may improve the general state of the immune system and reduce the activity of inflammatory processes.

**Figure 6:**
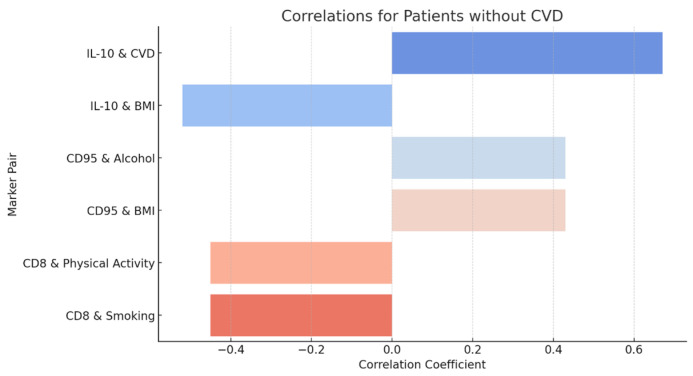
Correlations of immunological markers with clinical parameters in patients without CVD.

The graph in Figure 6 shows the results of correlation analysis between immunological markers and clinical parameters in patients without cardiovascular disease. The identified correlations illustrate the mechanisms of interaction between the immune system and metabolic and behavioral factors that can influence inflammatory and immune processes. The results of correlation analysis demonstrate the significance of immunological markers in assessing the influence of metabolic and behavioral factors on inflammatory processes in patients without cardiovascular disease. The identified correlations confirm the role of factors such as obesity, alcohol consumption, physical activity, and smoking in modulating the immune response.

**Figure 7:**
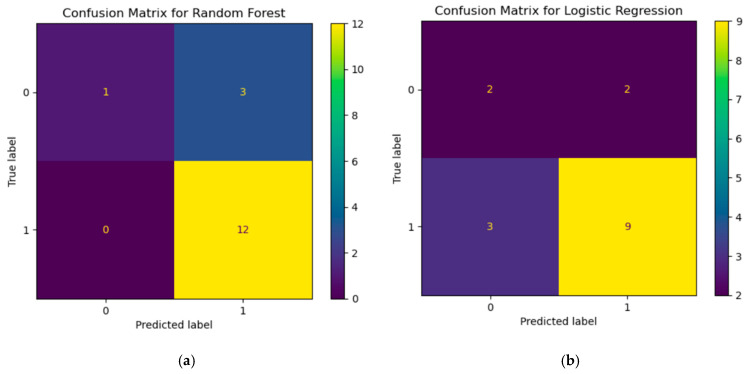
Confusion matrix (**a**) RF and (**b**) LR.

In the next step, machine learning methods (see Figure 7a,b) like random forest, logistic regression, and k-NN were used. Each of the models was trained on the data shown above. Separate experiments were conducted for each model with visualization of key metrics such as ROC curve and error matrix to evaluate the classification quality and identify the strengths and weaknesses of the approaches. All models were trained on the same data frame, which includes immunological data from patients with and without CVD.

We trained the model on patient characteristic data using the random forest method. The dataset contains labels 1 and 0, where 1 is a patient with CVD and 0 is a patient without CVD. The model trained on this data should make an immediate classification based on immunological parameters and deduce that the patient belongs to 1 or 0. The error matrix for this model shows that from class 0, the model was able to correctly classify only 1 case but made 3 errors assigning them to class 1. At the same time, for class 1, the model worked very well: it correctly predicted 12 cases and made no errors. This suggests that the random forest performs effectively in predicting class 1 but has difficulty with class 0, which may be due to unbalanced data or insufficient feature expression for class 0.

Using logistic regression, the error matrix shows that for class 0, the model correctly predicted 2 cases but made 2 errors. For class 1, 9 predictions were correct, and the model made 3 errors. Overall, logistic regression performs worse than random forest because it makes more errors for both classes. This may be because the model is linear and cannot capture complex relationships between traits, which limits its accuracy.

**Figure 8:**
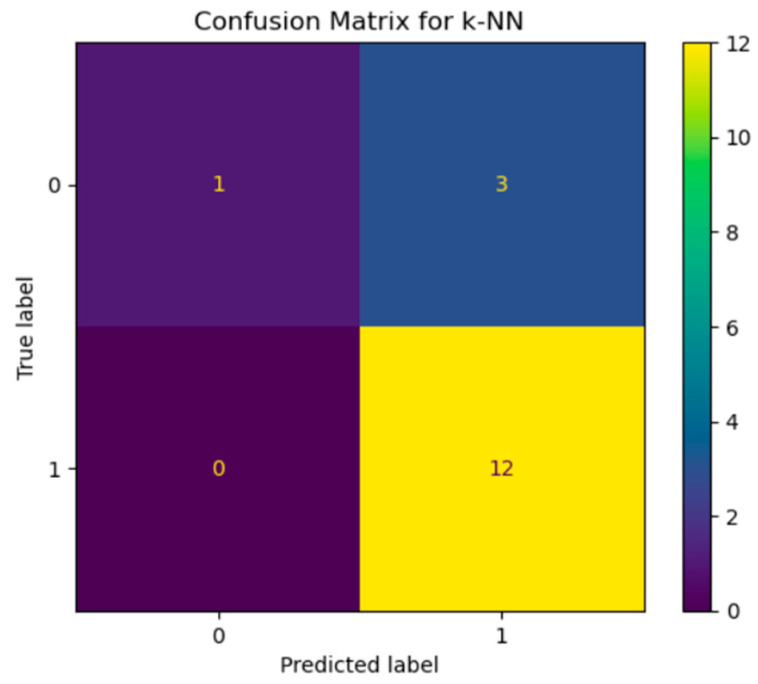
Confusion matrix of k-NN.

The k-nearest neighbors (k-NN) model showed similar results (see Figure 8) to the random forest. For class 0, it correctly classified 1 case but was wrong 3 times, assigning them to class 1. For class 1, the model predicted all 12 cases without error. This suggests that k-NN also performs well for class 1 but faces the same problem as the random forest: the difficulty of class 0 classification.

In general, all models are better at predicting class 1, but have difficulty with class 0. Perhaps the data contains class imbalance or class 0 features are not properly defined. In turn, data imbalance does have an impact in prediction. For example, a study [21] states that one common problem in multi-class and multi-label classification is class imbalance, where one or more classes make up a significant proportion of the total number of instances. This imbalance causes the neural network to prioritize features from the majority of classes during training, as their detection leads to higher scores.

**Figure 9:**
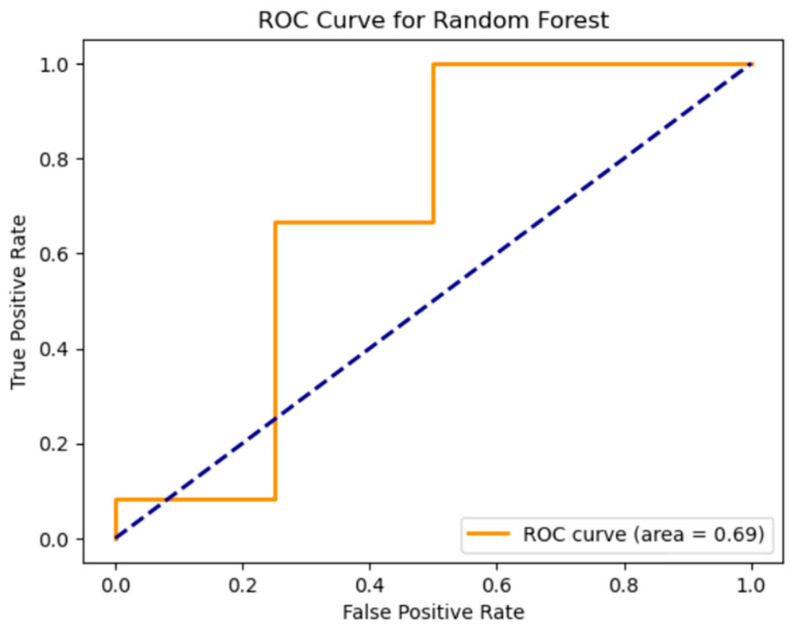
ROC curve for RF.

In Figure 9, random forest (RF) performed better as it eliminated the errors for class 1, but further analysis and model tuning are required to improve the classification of class 0.

**Figure 10:**
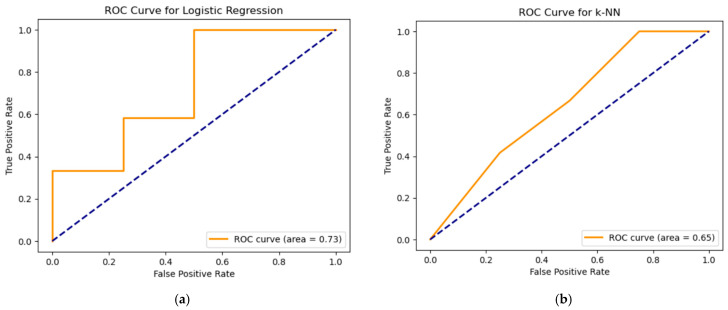
ROC curve for (**a**) LR and (**b**) k-NN.

To compare the different methods in Figure 10, we also analyzed the quality of the models using receiver operating characteristic curve (ROC) and area under the curve (AUC) values. Let us start by looking at the results of each model.

For random forest, the ROC curve shows that the AUC is 0.69, which means that the model has a moderate ability to discriminate between classes, but its performance is lower than we would like. An AUC of less than 0.7 indicates that the model is only slightly better than random guessing. Despite its good performance in classifying class 1 in the confusion matrix, the model faces difficulties in the overall balance of classification, especially considering class 0.

Logistic regression shows the best ROC curve with an AUC of 0.73. This figure indicates the better ability of the model to discriminate between classes compared to the random forest. This improvement is due to the linear nature of logistic regression, which can perform better on data with certain patterns even if the error matrix shows more errors for individual classes. In the study [22], logistic regression is noteworthy for the achieved R^2^: 0.82 scores in predicting hospital length of stay using machine learning on a large open medical dataset.

For the k-nearest neighbors (k-NN) model, the AUC is 0.65, which is the lowest score among all models. The ROC curve confirms that the model performs worse in classification compared to other approaches. This result is consistent with the confusion matrix where k-NN has difficulty in class 0 classification which reduces the overall performance. In a study [23] William DeGroat et al. used different machine learning techniques to detect biomarkers associated with cardiovascular diseases and predict them with high accuracy for precision medicine. Here the performance of k-NN was poor compared to RF, SVM, and XGBoost. The k-NN was a resource-intensive algorithm, providing the lowest performance with increased execution time compared to our previous classifiers.

Overall, logistic regression shows the greatest ability to discriminate between classes based on AUC data, but there is little difference between models.

**Figure 11:**
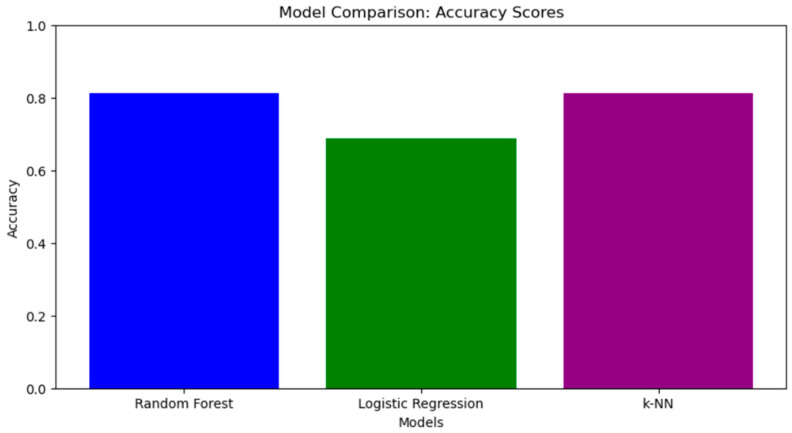
Accuracy for each model.

Figure 11 shows the accuracy values for random forest, logistic regression, and k-NN models. Random forest shows the highest accuracy, which confirms its ability to perform well on the classification task, especially for one of the classes. Also, in a study by Sohrab Effati [24], random forest algorithm was chosen for a web application due to its superior performance, achieving an accuracy of 99% for predicting hypertension and 97% for cardiovascular diseases, which outperforms the results of other models. Its high accuracy allows it to effectively assess workers’ individual risks and offer targeted recommendations to reduce morbidity.

The k-NN model has similar performance, indicating its competitiveness in this task where competitiveness is assessed using accuracy scores. Logistic regression shows the lowest accuracy among all the methods, which is consistent with its results from the error matrix and ROC curve. Now, comparing all these models, we decided to show you the result of random forest method.

**Figure 12:**
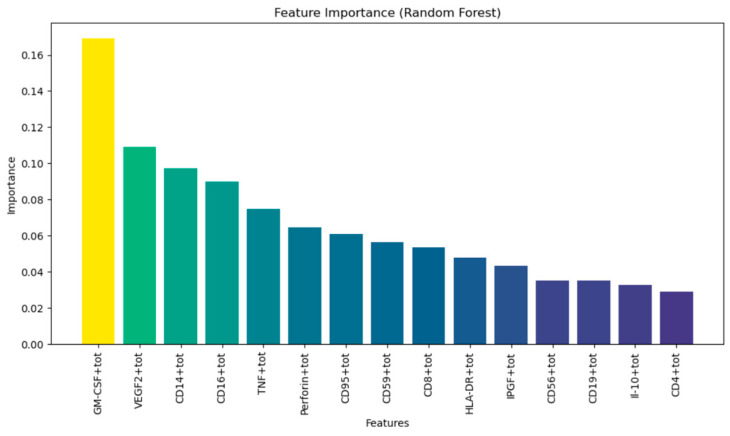
Feature importance (RF).

Figure 12 shows the importance of attributes in the random forest model for data analysis. The X-axis shows the attributes, and the Y-axis shows their importance. Importance is measured using the random forest model and reflects how much each attribute affects the prediction of the model. The attribute ‘GM-CSF’ has the highest importance, followed by ‘VEGF-2’, ‘CD14+’, and others, while attributes such as ‘CD8+’ and ‘Il-10’ rank last in importance. This helps in determining which features should be considered for model building and which can be excluded to improve its performance.

Since our models with RF, LR, and k-NN showed a maximum accuracy value of 0.81 and a maximum auction value of 0.69, we decided to improve the results and trained the model via XGBoost with the same clinical and immunological data. In further study, a predictive model for cardiovascular disease (CVD) detection was developed and trained using XGBoost gradient boosting and SMOTE method with high accuracy and robustness to multicollinearity of features.

Before training the model, a comprehensive preprocessing of the data was carried out. Due to the significant class imbalance, the SMOTE (synthetic minority over-sampling technique) technique was applied, which allows to artificially increase the number of examples of the minority class by generating synthetic samples based on the nearest neighbors. This approach allowed to offset the bias of the model towards the dominant class and improve its predictive ability.

**Figure 13:**
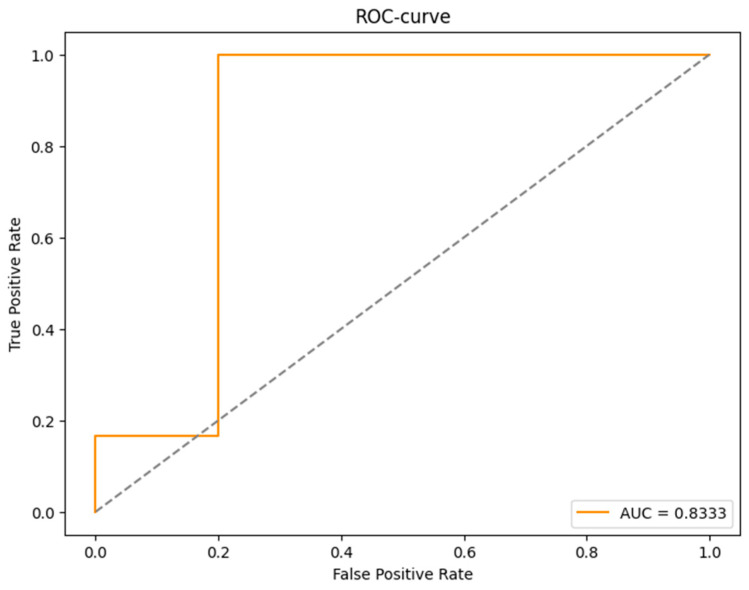
ROC curve (XGBoost).

Figure 13 shows the ROC curve illustrating the ratio of true positive to false positive classifications. The obtained value of AUC = 0.8333 indicates the high ability of the model to differentiate between patients with and without cardiovascular disease.

**Figure 14:**
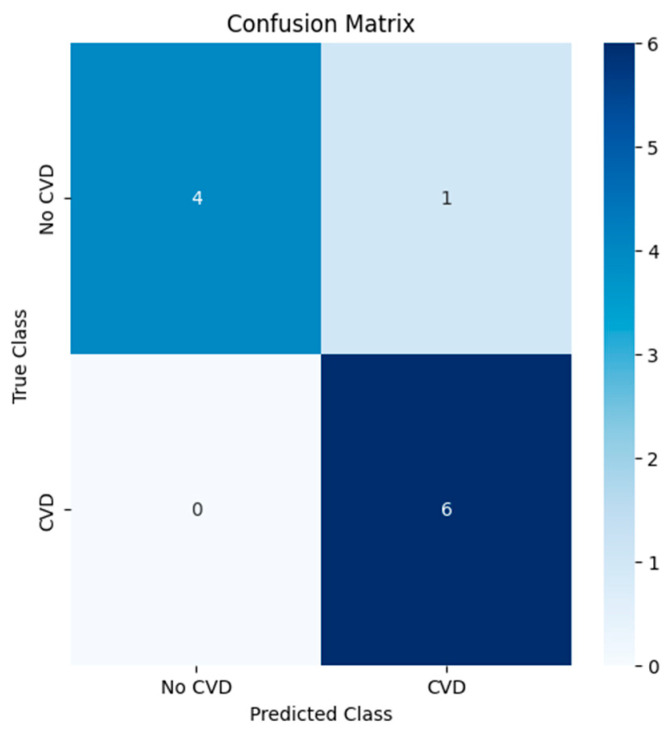
Confusion matrix (XGBoost).

It is worth noting that here the original dataset was divided into training (80%) and test (20%) samples, taking into account the stratification by target class. And the error matrix shows (Figure 14) that the model correctly classified 10 out of 11 cases: 4 cases without CVD (no CVD) and 6 cases with CVD. There was 1 false positive prediction when the model incorrectly identified the absence of CVD as presence.

**Figure 15:**
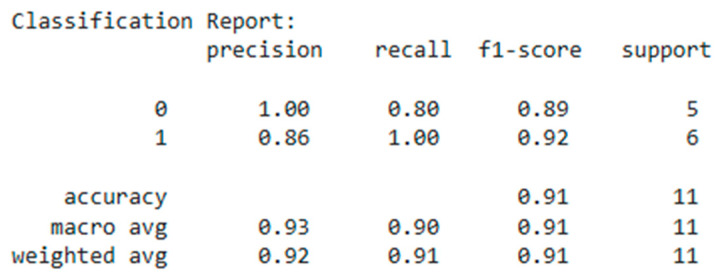
Metrics (XGBoost).

In Figure 15 the model demonstrated an overall accuracy of 91%, indicating high predictive ability. The model is balanced and demonstrates high accuracy and F1-score (~0.91), but class 0 recognition could be improved to reduce false positives.

**Figure 16:**
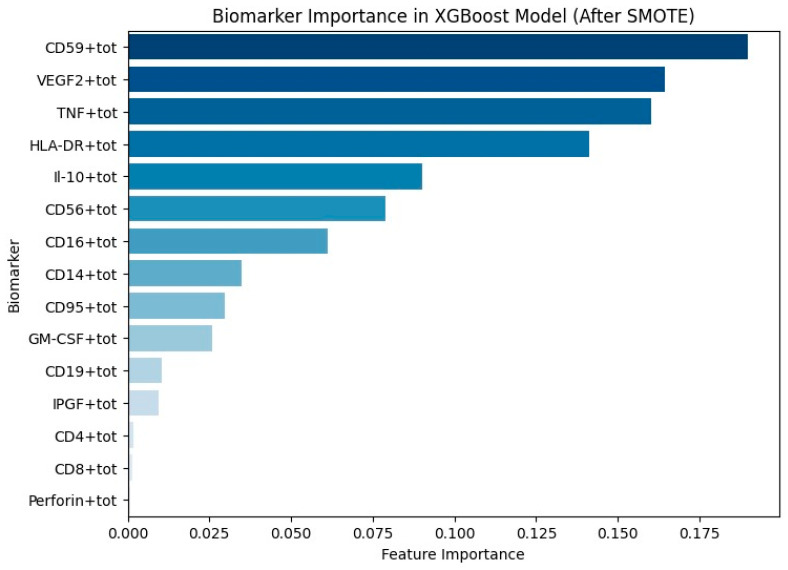
Biomarker importance (XGBoost).

Figure 16 shows which immunological markers contribute most to the prediction of premature cardiovascular aging using the XGBoost model. CD59+ is the most significant biomarker as this protein is involved in protecting cells from immune system damage. VEGF-2 and TNF-α are important predictors: VEGF-2 is associated with new vessel formation and its excess may be associated with inflammation; TNF-α (tumor necrosis factor) plays a key role in inflammatory processes. High levels may indicate chronic inflammation associated with CVD (cardiovascular disease) risk.

The results of the model testing demonstrated high prediction accuracy. The accuracy value was 0.9091%, indicating high correctness of patient classification. The AUC metric of 0.8333 confirms that the model has a high ability to distinguish between patients with and without CVD compared to RF, LR, and k-NN. Other researchers also argue the effectiveness of the XGBoost method for predicting SWD. Salah. H and other co-authors in a study found that the XGBoost model showed AUC-ROC: 84.5% among other models [25], and in Han S., the results showed that XGBoost has high AUC-ROC among other models: 75% [26]. It can be said that our XGBoost model with AUC-ROC = 83% shows its reliability.

## 5. Discussion

This study looks powerful because it combines immunological, clinical, and behavioral aspects. The construction of correlation matrices gives a clear picture of the relationships between parameters such as BMI, smoking, cardiovascular disease, and immune markers. For example, the positive correlation between HLA-DR and BMI emphasizes the activation of antigen-presenting cells in overweight people, while the negative correlation of IL-10 with BMI demonstrates a decrease in anti-inflammatory mechanisms in the same group. This is quite logical given that obesity is often accompanied by chronic inflammation.

Based on the correlation results, we can see some interesting facts about the influence of lifestyle. Smoking, for example, is associated with suppression of CD8+ T-lymphocytes, which clearly has a negative impact on the adaptive immune response. And the link between education level and HLA-DR expression seems unexpected at first glance, but if you think about it, it may be related to lifestyle and access to medical resources.

Most importantly, the paper used machine learning techniques to analyze the data, which is an integral part when dealing with data these days. Random forest showed the best results for classification, especially for one of the classes. But it is seen that the models face the problem of data imbalance. This is a fairly common problem, and it is probably worth trying balancing techniques like oversampling or using more complex algorithms like gradient boosting.

What is a bit disconcerting is that the results of the models do not always look unambiguous. For example, the AUC of the random forest—0.69—is only slightly higher than the random guess. This suggests that perhaps the data is not expressive enough or the signs need to be refined.

The results demonstrate that the use of machine learning methods, particularly XGBoost can effectively predict cardiovascular diseases based on clinical and immunological data. The use of SMOTE for class balancing improves the quality of predictions, and the identified significant biomarkers may serve as a basis for further research in the field of pathogenesis and early diagnosis of CVD.

Either way, the work provides a strong foundation for further research. It would be great to delve deeper into the dynamics of changes in immune markers to understand their long-term effects. And another promising direction could be the development of an integrative model that combines biological and behavioral data to more accurately predict the risks of aging. Overall, the study emphasizes the importance of an integrated approach and the use of modern technologies to study such complex processes as aging.

## 6. Conclusions

This paper presents an analysis of the links between immunological, clinical, and behavioral factors, revealing key mechanisms of premature aging and related diseases.

The results confirm that systemic inflammation, reflected by markers CD14+, HLA-DR, and IL-10, plays a central role in the pathogenesis of aging. The positive correlation of HLA-DR with BMI (r = 0.39) confirms that obesity is accompanied by activation of antigen-presenting cells and increased inflammation. At the same time, the negative correlation of IL-10 with BMI (r = −0.52) emphasizes the reduction of anti-inflammatory mechanisms in obesity, which contributes to metabolic disorders. The negative correlation of smoking with CD8+ level (r = −0.4) indicates suppression of adaptive immune response by bad habits.

The use of artificial intelligence methods (random forest, logistic regression, k-nearest neighbors, XGBoost) to diagnose cardiovascular disease and early aging gave good results. Among all the models, XGBoost performed the best, achieving AUC = 0.8333, which demonstrates its high predictive ability. Random forest also showed high accuracy, but was slightly inferior to XGBoost. In turn, logistic regression demonstrated AUC = 0.73 and K-NN method—AUC = 0.65, which confirms the potential of machine learning in the diagnosis of cardiovascular diseases.

The study highlights the importance of a multidisciplinary approach that integrates immunological, social, and behavioral aspects. The identified links provide a basis for the development of biomarkers for early prognosis, new treatments, and preventive strategies.

The findings have implications for basic and personalized medicine, expanding the understanding of the mechanisms of aging. Further research should focus on studying the interaction between the immune system and metabolism, creating a mathematical model of aging and developing a model with an accuracy higher than 95 percent.

## Data Availability

The original contributions presented in this study are included in the article. Further inquiries can be directed to the corresponding author.
